# Untargeted Metabolomics of Fermented Rice Using UHPLC Q-TOF MS/MS Reveals an Abundance of Potential Antihypertensive Compounds

**DOI:** 10.3390/foods9081007

**Published:** 2020-07-27

**Authors:** Eric Banan-Mwine Daliri, Fred Kwame Ofosu, Ramachandran Chelliah, Joong-Hark Kim, Jong-Rae Kim, Daesang Yoo, Deog-Hwan Oh

**Affiliations:** 1 Department of Food Science and Biotechnology, College of Agriculture and Life Sciences, Kangwon National University, Chuncheon 24341, Gangwon-do, Korea; ericdaliri@yahoo.com (E.B.-M.D.); fkofosu@gmail.com (F.K.O.); ramachandran@gmail.com (R.C.); jrkim@gmail.com (J.-R.K.); dsyoo@yahoo.com (D.Y.); 2 Department of Medical Biotechnology, College of Biomedical Sciences, Kangwon National University, Chuncheon 24341, Gangwon-do, Korea; jhk@yahoo.com; 3 R&D, Erom, Co., Ltd., Chuncheon 24427, Gangwon-do, Korea; 4 R&D, Hanmi Natural Nutrition Co., LTD 44-20, Tongil-ro 1888 beon-gil, Munsan, Paju 10808, Gyeonggi, Korea; 5 R&D, H-FOOD, 108-66, 390 gil, Jingun Oh Nam-Ro, Nam Yang, Ju-Shi 12041, Gyung Gi-Do, Korea

**Keywords:** bioactive peptides, hypertension, phenolic compounds, functional food

## Abstract

Enzyme treatment and fermentation of cereals are known processes that enhance the release of bound bioactive compounds to make them available for bioactivity. In this study, we tested the angiotensin converting enzyme (ACE) inhibitory ability of destarched rice, Prozyme 2000p treated destarched rice (DP), and fermented DP samples. Prozyme 2000p treatment increased the ACE inhibitory ability from 15 ± 5% to 45 ± 3%. Fermentation of the Prozyme 2000p treated samples with *Enterococcus faecium* EBD1 significantly increased the ACE inhibitory ability to 75 ± 5%, while captopril showed an ACE inhibition of 92 ± 4%. An untargeted metabolomics approach using Ultra-high-performance liquid tandem chromatography quadrupole time of flight mass spectrometry revealed the abundance of vitamins, phenolic compounds, antioxidant peptides, DPP IV inhibitory peptides, and antihypertensive peptides in the fermented samples which may account for its strong ACE inhibition. Although fermented DP had decreased fatty acid levels, the amount of essential amino acid improved drastically compared to destarched rice. Our results show that fermenting Prozyme-treated destarched rice with *Enterococcus faecium* EBD1 generates abundant bioactive compounds necessary for developing antihypertensive functional foods.

## 1. Introduction

In recent years, cereal grains such as rice have attracted much scientific attention [1] because they contain phenolic compounds such as quercetin, ferulic acid, and salicylic acid [2] which have strong antioxidant abilities [3]. Over the years, oxidative stress has been shown to play a key role in the pathogenesis of hypertension [4,5] since reactive oxygen species can cause endothelial dysfunction [6] leading to arterial stiffness in humans [7,8]. One popular physiological mechanism of hypertension is the renin-aldosterone-angiotensin system (RAAS) [9]. In the RAAS, renin cleaves angiotensinogen to release angiotensin I which is then hydrolyzed by angiotensin 1-converting enzyme (ACE) to generate angiotensin II (a vasoconstrictor) [10]. Interestingly, phenolic compounds have been shown to strongly inhibit angiotensin 1-converting enzyme (ACE) [11,12] and the strength of inhibition is directly proportional to the number of hydroxyl groups they possess [13]. In addition to polyphenols, rice contains proteins which when hydrolyzed, generate bioactive peptides [10]. Many potent antihypertensive peptides such as IHRF [14], VNP, and VWP [15] have been identified in rice. These peptides may reduce hypertension by inhibiting ACE and/or renin activities [16], triggering nitric oxide production, or blocking angiotensin II receptors [17]. Antihypertensive peptides are usually generated in foods either by enzyme hydrolysis or fermentation of protein samples. Lactic acid bacteria have been effective in fermenting food materials to release polyphenols [18] and to generate antihypertensive peptides [19]. In recent years, metabolomic techniques such as ^1^H-NMR (^1^H-nuclear magnetic resonance), GC-MS (gas chromatography-mass spectrometry), and LC-MS (liquid chromatography-mass spectrometry) have been used to identify metabolites in foods [20]. Among the techniques commonly used, LC-MS is the most used in metabolomic studies due to its detection sensitivity, high resolution, and nonderivatization of samples [21]. Ultra-high-performance liquid tandem chromatography quadrupole time of flight mass spectrometry (UHPLC-QTOF/MS) is a new approach in chromatography and was developed based on LC-MS to determine and quantify more metabolites [22]. In this study, we determined the ACE inhibitory ability of destarched rice before and after Prozyme 2000p treatment and after fermentation. We then used UHPLC-QTOF/MS to analyze the metabolite changes that occurred after Prozyme 2000p treatment and after fermentation to identify bioactive compounds generated by the processing methods.

## 2. Materials and Methods

### 2.1. Chemicals and Cultures

All chemical reagents were of analytical grade. ACE-1 Assay Kit (Fluorometric) was purchased from Biovision (Milpitas, CA 95035, USA). All other reagents, unless specified, were purchased from Sigma-Aldrich, Seoul, Korea. Two rice powder samples (*Oriza sativa* L. variety Japonica) were received from Erom Company Limited (Chuncheon-si, Kangwon-do, Korea). One sample (destarched rice) was composed of rice powder treated with α-amylase to hydrolyze the starch present. The second sample (destarched rice + Prozyme (DP)) consisted of destarched rice treated with Prozyme 2000p.

*Enterococcus faecium* EBD1 was obtained from the Department of Food Science and Biotechnology, Kangwon National University, Korea to be used for fermentation. This bacterium was used in the current study because it demonstrated strong proteolytic ability in our previous study (data not shown). The bacteria stock culture was stored at −80 °C in de Man, Rogosa and Sharpe (MRS) broth (Difco), containing 20% glycerol (*v/v*). The bacterium was spread on MRS agar and incubated at 37 °C overnight to obtain single colonies. MRS broth (10 mL) was then inoculated with a single bacteria colony and incubated at 37 °C. The cells were harvested at the exponential phase of growth. The number of viable bacterial cells was determined by the plate count on MRS agar.

### 2.2. Rice Fermentation

The bacteria growth medium for the lactic acid bacteria consisted of 10% (*w/v*) Prozyme 2000p treated destarched rice powder in distilled water. The growth media was sterilized by autoclaving at 121 °C for 15 min before inoculating with lactic acid bacteria. *E. faecium* EBD1 (2 × 10^8^ cfu/mL) was transferred from an overnight culture to a 200 mL of autoclaved growth media (pH 6). The media was incubated at 37 °C with 150 rpm agitation for 48 h. The media was then centrifuged at 10,000× *g* for 10 min and the supernatant was freeze-dried using TFD5505 table top freeze dryer (ilshinBioBase Co. Ltd., Gyeonggi-do, Korea) and the dried samples were stored at −20 °C for further analysis. The final fermented product was labelled as Fermented DP.

### 2.3. Determination of Angiotensin 1-Converting Enzyme Inhibitory Ability

ACE inhibitory activities of destarched rice, Prozyme treated distarched rice (DP), and fermented Prozyme-treated rice (fermented DP) powder were measured using an ACE-1 Assay Kit according to the manufacture’s instructing with some modifications (Table S1). Briefly, 10 µL of diluted ACE-1 solution was transferred to a 96 well plate and the volume was adjusted to 50 µL/well with ACE-1 assay buffer. An aliquot of the rice samples (20 µL, 5 mg/mL) was added to the wells and mixed thoroughly. Captopril (20 µL, 5 mg/mL) was used as a positive control. ACE-1 substrate (50 µL) was added to the wells and mixed. Fluorescence (Ex/Em = 330/430 nm) was measured in a kinetic mode for 2 h at 37 °C. A standard curve was prepared using the Abz-standard solution. The ACE1 activity was calculated as:

ACE1 activity = B×D/(ΔT×P) = pmol/minute/mg,

where:

B = Abz in sample based on standard curve slope (pmol),

ΔT = reaction time (minutes),

P = sample used into the reaction well (in mg),

D = sample dilution factor.

One unit of ACE-1 activity is the amount of enzyme that catalyzes the release of 1 nmol of Abz per min from the substrate under the assay conditions at 37 °C. The extent of inhibition was calculated as 100% × [(B − A)/B] where A is the ACE-1 activity in the presence of ACE and ACE inhibitor, B is the ACE-1 activity without ACE inhibitory component.

### 2.4. Metabolomics Analysis

Each rice sample (1 g) was extracted with 20 mL of 50% methanol and placed on a mini rocker (Clinical Diagnostics, Gangnam, Korea) overnight. The samples were mixed completely for 30 s using a vortex and subsequently centrifuged at 12,000× *g* for 12 min at 4 °C. Aliquots (1 mL) of the supernatants were filtered through 0.25 µm pore size Millex syringe filters (Merck KGaA, Darmstadt, Germany) and transferred into LC-MS vials. LC-MS/MS analysis was carried out using a UHPLC (SCIEX ExionLC AD system, Framingham, MA, USA) connected to a controller, a pump, a degasser, an autosampler, column oven, and a photodiode array detector (ExionLC) coupled to a quadrupole time-of-flight mass spectrometer (Q-TOF-MS) (X500R QTOF). The analytical column used was a 100 × 3 mm, Accucore C18 column (Thermo Fisher Scientific, Waltham, MA, USA). Solvent A consisted of water with 0.1% formic acid and solvent B was methanol. The chromatography was carried at a flow rate of 0.4 mL/min. A linear gradient was programmed for 25 min as follows: 0–3.81 min, 9% to 14% B; 3.81–4.85 min, 14% to 15% B; 4.85–5.89 min, 15% B; 5.89–8.32 min, 15% to 17% B; 8.32–9.71 min, 17% to 19% B; 9.71–10.40 min, 19% B; 10.40–12.48 min, 19% to 26% B; 12.48–13.17 min, 26% to 28% B; 13.17–14.21 min, 28% to 35% B; 14.21–15.95 min, 35% to 40% B; 15.95–16.64 min, 40% to 48% B; 16.64–18.37 min, 48% to 53% B; 18.37–22.53 min, 53% to 70% B; 22.53–22.88 min, 70% to 9% B; 22.88–25.00 min, 9% B. The injection volume was 5 μL. The Q-TOF-MS was set for the negative mode through a mass range of 100–1000 and a resolution of 5000. The capillary and cone voltages used to record full mass spectra were 1.45 kV and 30 V, respectively. The flow rate of Helium (the cone gas) was 45 L/h, while the flow rate of the desolvation gas (N_2_) was 900 L/h. The temperature of N_2_ was 250 °C, the ion source temperature was 120 °C, while the collision energies needed to obtain the MS/MS spectra were set at 15, 20, and 30 V.

### 2.5. Data Analysis

All data were obtained and processed using SCIEX OS 1.0 software. A non-target algorithm was used for peak finding. Matrix and sample specific signals were separated from true contaminations using an automatic sample-control comparison algorithm. Compound identification was done by using empirical formula finding, MS/MS library searching, and online database searching. Compound names, peak area, retention time, similarity to metabolites in the database, and mass (*m/z*) were ultimately imported into Microsoft Excel.

### 2.6. Statistical Analysis

For ACE inhibitory ability tests, all experiments were carried out in triplicates and the results were expressed as mean ± standard deviation. The statistical analysis of data was performed using GraphPad Prism 5.0 (2007) statistical software system (GraphPad Software Inc., San Diego, CA 92037 USA). *p* < 0.05 was considered significant as evaluated by Student’s *t*-test.

ClustVis software (http://biit.cs.ut.ee/clustvis/) was used in multivariate statistical analyses, including principal component analysis (PCA) and heat maps [23]. PCA was performed to visualize the changes in metabolite composition in destarched rice, Prozyme treated destarched rice (DP), and fermented DP. Heat maps and PCA plots were drawn by using identified compounds and their peak area. We used peak areas because it is widely accepted that the peak area of an analyte in a chromatograph is directly proportional to its concentration [24,25,26].

## 3. Results and Discussion

Rice consists of about 90% carbohydrate, up to 8% protein, and about 2% fat [27]. These major components are bound together in the food matrix and hence, hydrolyzing rice starch with α-amylase enables the release of bound proteins and other biomolecules from the food matrix, making them available for subsequent reactions.

### 3.1. ACE Inhibitory Activity of Rice Samples

**Figure 1:**
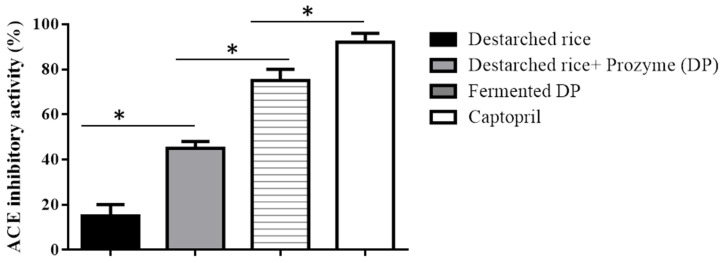
ACE inhibitory activities of destarched rice, Prozyme treated destarched rice (DP), and fermented DP. Data show mean SD (*n* = 3). Each bar represents the means of three replicates ± S.D. * denotes a significant difference between the ACE inhibitory ability of the samples.

Prozyme treatment significantly (*p* < 0.05) increased the ACE inhibitory ability of destarched rice from 15 ± 5% to 45 ± 3% (Figure 1). This could be due to the release of ACE inhibitory compounds from the rice proteins during Prozyme proteolysis. Our observation agrees with earlier studies that showed that hydrolysis of rice proteins enhances the generation of ACE inhibitory hydrolysates [28]. Subsequent fermentation of the Prozyme hydrolysate with *E. faecium* EBD1 further increased the inhibitory effect up to 75 ± 5%, while Captopril (a standard ACE inhibitory peptide) demonstrated a 92 ± 4% inhibitory ability.

**Figure 2:**
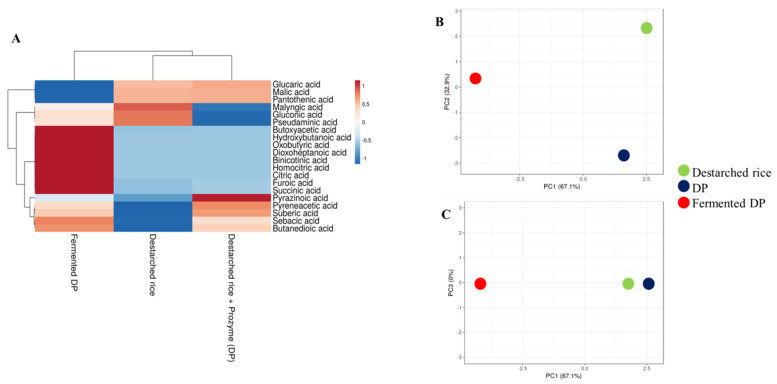
Relative levels of organic acids and volatile compounds in destarched rice, Prozyme treated destarched rice (DP), and fermented DP. (**A**) Heat map shows the different levels of organic acids present in the three samples. The color range from red to blue represents higher to lower levels of organic acids. (**B**,**C**) are principal component analysis (PCA) plots. (**B**) consists of PC1 and PC2, while (**C**) consists of PC1 and PC3. Red circles represent fermented DP, black represents Prozyme treated destarched rice (DP), and green represents destarched rice samples.

During lactic acid bacteria fermentation, a number of organic acids are generated from the sugars and lipids available in the growth media and this affects the flavor and pH of the final product [29]. ACE activity is best at a pH of ~8.3 [11] and hence, the increased acidity of fermented food may contribute to inhibition of the enzyme. In this study, a total of twenty organic acids were detected in all the rice samples (Figure 2A). Ten organic acids were detected in destarched rice, 14 in DP, and 19 in fermented DP (Table S2). Only the fermented samples contained homocitric acid, citric acid, binicotinic acid, dioxoheptanoic acid, oxobutyric acid, and hydroxybutanoic acid. Meanwhile, butyric acid [30], nicotinic acid [31], and citric acid are known antihypertensive compounds and hence, their enrichment in the fermentate makes it a potential antihypertensive functional food. Although the types and levels of organic acids in the three samples were different (Figure 2B), organic acid profiles of destarched rice and DP were similar (Figure 2C).

### 3.2. Amino Acid Levels in the Rice Samples

**Figure 3:**
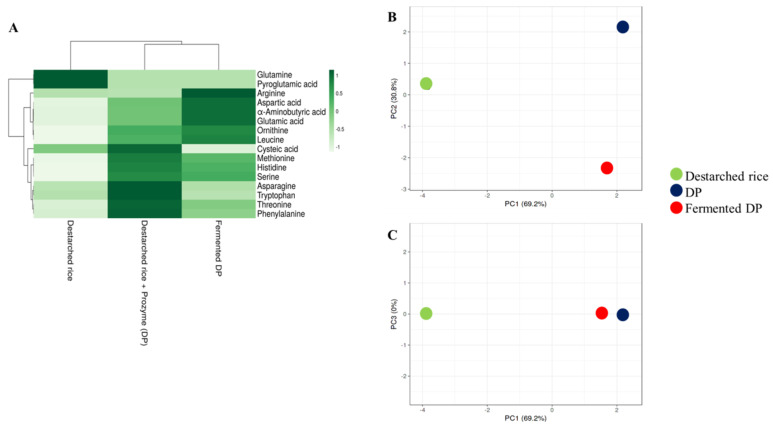
Relative levels of amino acids in destarched rice, Prozyme treated destarched rice (DP), and fermented DP. (**A**) Heat map shows the different levels of organic acids present in the three samples. The color range from green to white represents higher to lower levels of amino acids. (**B**,**C**) are principal component analysis (PCA) plots. (**B**) consists of PC1 and PC2, while (**C**) consists of PC1 and PC3. Red circles represent fermented DP, black represents Prozyme treated destarched rice (DP), and green represents destarched rice samples.

A total of sixteen amino acids were detected in the analyzed samples (Figure 3A, Table S3). In this study, destarched rice contained the least free amino acids contents since most of the amino acids might have remained bound to their parent proteins. Hydrolysis of rice proteins with Prozyme resulted in the cleavage and release of high amounts of essential amino acids such as phenylalanine, threonine, methionine, histidine, and tryptophan (Figure 3). The levels of these essential amino acids reduced drastically after fermentation except for histidine and methionine. Glutamine, cysteic acid, tryptophan, and pyroglutamic acid, though present in the Prozyme treated sample, were not detected in the fermented sample (Table S3) probably because they were consumed by the bacteria to meet their nitrogen requirements during the fermentation process. Meanwhile, the level of leucine (an essential amino acid) and some conditionally essential amino acids such as ornithine, arginine, and serine were increased after fermentation (Figure 3). As shown in the PCA plot (Figure 3B), the amino acid profiles of DP were similar to those of fermented DP but different from destarched rice (Figure 3C).

### 3.3. E. faecium EBD1fermentation Increases the Amount of Phenolic Compounds in Rice

**Figure 4:**
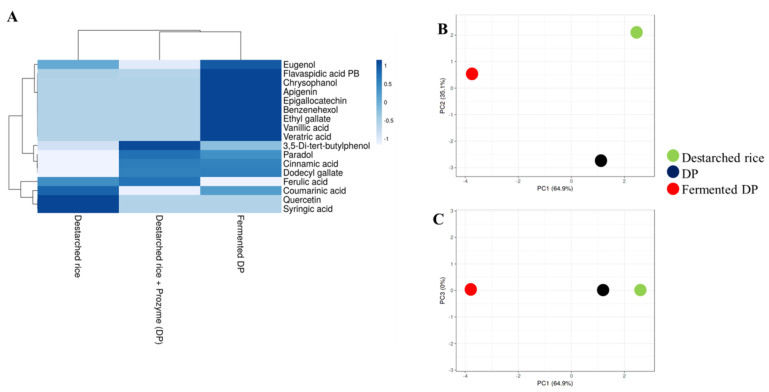
Relative levels of phenolic compounds in destarched rice, Prozyme treated destarched (DP), and fermented DP. (**A**) Heat map shows the different levels of phenolic compounds present in the three samples. The color range from blue to white represents higher to lower levels of phenolic compounds. (**B**,**C**) are principal component analysis (PCA) plots. (**B**) consists of PC1 and PC2, while (**C**) consists of PC1 and PC3. Red circles represent fermented DP, black represents Prozyme treated destarched rice (DP), and green represents destarched rice samples.

Recent studies have shown that a strong hydrophobic interaction exists between phenolic compounds and other rice components [32]. In this study, 10 out of the 17 detected phenolic compounds were present in destarched rice, while 9 were detected after Prozyme treatment (Figure 4, Table S4). In the fermented samples however, 16 phenolic compounds were present and this implies that the fermentation process effectively enhanced the liberation of phenolic compounds that remained bound to the rice matrix even after α-amylase and Prozyme treatments. Fermentation resulted in the enrichment of strong antioxidant and antihypertensive phenolic compounds such as vanillic acid [33], eugenol [34], veratric acid [35], ethyl gallate [36], epigallocatechin [37], apigenin [38], and chrysophanol [39]. Our observation agrees with an earlier study that demonstrated that microbial fermentation increases the polyphenol contents of cereals [40,41].

### 3.4. E. faecium EBD1fermentation Reduces Lipid Levels in Rice

**Figure 5:**
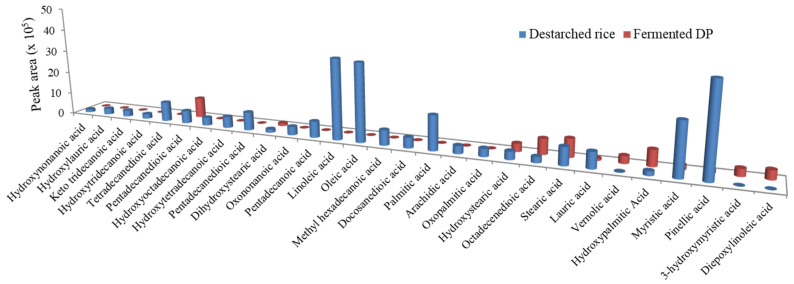
The relative levels of fatty acids in destarched rice and fermented DP.

Oxidation of lipids during fermentation results in the generation volatile compounds such as aldehydes and alcohols which contribute to flavor [42]. In this study, *E. faecium* EBD1fermentation caused a general reduction in the fatty acid levels in the rice sample (Figure 5). The levels of stearic acid (an antioxidant fatty acid) [43] slightly increased, while lauric acid (an antihypertensive fatty acid) [44] was still detectable in the fermentate. Our results are different from an earlier study which found no significant changes in lipid content during millet fermentation [45].

### 3.5. E. faecium EBD1fermented Rice Is Enriched with Antihypertensive Peptides

Lactic acid bacteria possess cell envelop proteinases that hydrolyze proteins in media into oligopeptides after which they are absorbed to meet their nutritional needs [9]. For this reason, we analyzed the peptides generated in the fermented samples and determined their potential functions by comparing them with similar peptides already reported in literature (Table 1). In all, 32 peptides were identified in the fermented rice samples among which 16 are reported in literature as antihypertensive peptides. Nine peptides had their carboxyl or amino-terminal amino acid sequences similar to reported antihypertensive peptides, while 3 peptides, namely VPL [46], MV, and HR [47] were found to be dipeptidyl peptidase-4 (DPP IV) inhibitors. Many studies have shown the ability of DPP IV inhibitors to reduce hypertension [48,49]. One antioxidative peptide EL, was also found in the peptide profile of the fermented samples. The presence of these bioactive peptides in the fermented sample may contribute to the strong ACE inhibitory activity observed in this study. Meanwhile, functional analysis is required to confirm the antihypertensive activity of peptides in the fermented sample that contain sequences of already known antihypertensive peptides at their C and N terminals. Our findings are similar to other studies that reported that phenolic compounds [50,51] and bioactive peptides [52] in food can inhibit ACE activity.

## 4. Conclusions

Using untargeted metabolomics, we found that Prozyme treatment and subsequent fermentation enhanced the generation of organic acids (flavor compounds) and essential amino acids. The fermented DP samples also contained known antihypertensive phenolic compounds as well as antihypertensive peptides which make the sample a promising material for developing cheap antihypertensive foods. Despite the antihypertensive potential of the fermented product, further studies regarding the ability of *Enterococcus faecium* EBD1 fermented Prozyme-treated destarched rice to reduce high blood pressure in vivo is warranted.

## Figures and Tables

**Table 1:** Peptides identified in *Enterococcus faecium* EBD1 fermented DP.

Retention Time	Peak Area	Adduct/Charge	Precursor Mass	Found at Mass	Formula Finder Results	Peptides	Similar Peptides Reported in Literature*	Peptide Function	Reference
0.93	221,600	[M-H]-	227.116	227.115	C9H16N4O3	GPG	**GPG**	Antihypertensive	[53]
0.97	1,227,000	[M-H]-	516.206	516.2053	C19H31N7O10	QQQD	**LQQQ**	Antihypertensive	[54]
1.31	506,800	[M-H]-	185.058	185.0572	C7H10N2O4	EG	**EG**	Antihypertensive	[55]
3.16	501,500	[M-H]-	419.121	419.1206	C18H20N4O8	GGG	**GGG**	Antihypertensive	[56]
8.22	292,200	[M-H]-	442.232	442.2311	C19H33N5O7	PKEA	**VDKEA**	Antihypertensive	[57]
8.4	219,500	[M-H]-	426.237	426.2363	C19H33N5O6	GVGVP	Not found	-	-
8.63	2,611,000	[M-H]-	227.105	227.1039	C10H16N2O4	EV	**EV**	Antihypertensive	[58]
9.23	1,306,000	[M-H]-	291.1	291.0989	C14H16N2O5	EY	**EY**	Antihypertensive	[59]
9.48	192,900	[M-H]-	544.228	544.2266	C23H31N9O7	WGGGGGG	**GGG**	Antihypertensive	[56]
9.87	10,460	[M-H]-	293.116	293.1143	C14H18N2O5	DF	**DF**	Antihypertensive	[60]
10.29	210,900	[M-H]-	326.209	326.2087	C16H29N3O4	VPL	**VPL**	DPP IV inhibitor	[46]
10.6	1,264,000	[M-H]-	383.231	383.2305	C18H32N4O5	PLG	**PLG**	Antihypertensive	[61]
11.56	195,100	[M-H]-	453.201	453.1996	C20H30N4O8	PDGA	Not found	-	-
11.61	3,821,000	[M-H]-	241.121	241.1196	C11H18N2O4	EL	**EL**	Antioxidative	[62]
11.81	432,500	[M-H]-	489.273	489.2725	C25H38N4O6	VLPY	**VLPYP**	Antihypertensive	[63]
11.84	243,600	[M-H]-	518.227	518.2263	C24H33N5O8	DISW	**ISW**	Antihypertensive	[64]
11.91	478,100	[M-H]-	395.195	395.1941	C18H28N4O6	YLS	**YL**	Antihypertensive	[65]
11.92	267,600	[M-H]-	319.135	319.1335	C13H24N2O5S	MG	**MG**	Antihypertensive	[66]
12.12	249,800	[M-H]-	473.209	473.2079	C20H34N4O7S	EMVP	**MMVPI**	Antihypertensive	[67]
12.94	1,020,000	[M-H]-	489.237	489.2361	C24H34N4O7	GPLA	**GPL**	Antihypertensive	[68]
13.0	471,200	[M-H]-	774.334	774.3331	C44H49N5O6S	AW	**AW**	Antihypertensive	[69]
13.3	187,800	[M-H]-	180.974	180.9733	C2H2N2O8	GG	**GG**	Antihypertensive	[58]
13.41	1,627,000	[M-H]-	496.316	496.3145	C24H43N5O6	IVPVA	**KPVAL**	Antihypertensive	[70]
13.42	1,415,000	[M-H]-	528.284	528.2836	C27H39N5O6	FPIV	**GPFPIIV**	Antihypertensive	[70]
14.17	1,171,000	[M-H]-	510.331	510.3303	C25H45N5O6	GGGGG	**GGG**	Antihypertensive	[56]
14.24	409,400	[M-H]-	482.263	482.2627	C22H37N5O7	VVPQ	**VPQ**	Antihypertensive	[71]
15.88	153.2	[M-H]-	743.35	743.3477	C33H56N6O9S2	VMV	**MV**	DPP IV inhibitor	[47]
17.11	14,500	[M-H]-	267.067	267.0646	C8H16N2O6S	CE	Not found	-	-
17.22	1,126,000	[M-H]-	310.165	310.1637	C12H21N7O3	HR	**HR**	DPP IV inhibitor	[47]
17.8	360,400	[M-H]-	457.169	457.1688	C17H34N2O8S2	CC	**CCD**	Antihypertensive	[72]
21.12	43,240	[M-H]-	474.264	474.2634	C22H41N3O6S	ML	**RML**	Antihypertensive	[73]
22.19	29,960	[M-H]-	473.284	473.2837	C19H38N8O6	KTAR	**KTAP**	Antihypertensive	[63]

*Boldened alphabets represent amino acids sequences identified in this study and previous studies.

## Supplementary Materials

The following are available online at https://www.mdpi.com/2304-8158/9/8/1007/s1. Table S1: Procedure for assay of ACE activity. Table S2: Organic acids and volatile compounds in destarched rice, Prozyme treated destarched rice (DP) and fermented DP. Table S3: Amino acids in destarched rice, Prozyme treated destarched rice (DP) and fermented DP. Table S4: Phenolic compounds in destarched rice, Prozyme treated destarched rice (DP) and fermented DP.

## References

[1] Zhu F. (2018). Anthocyanins in cereals: Composition and health effects. Food Res. Int..

[2] Das A.J., Das G., Miyaji T., Deka S.C. (2016). In vitro antioxidant activities of polyphenols purified from four plant species used in rice beer preparation in Assam India. Int. J. Food Prop..

[3] Stagos D. (2020). Antioxidant activity of polyphenolic plant extracts. Antioxidants.

[4] Qu W., Du G.-L., Feng B., Shao H. (2019). Effects of oxidative stress on blood pressure and electrocardiogram findings in workers with occupational exposure to lead. Int. J. Med. Res..

[5] Montezano A.C., Touyz R.M. (2012). Molecular mechanisms of hypertension—Reactive oxygen species and antioxidants: A basic science update for the clinician. Can. J. Cardiol..

[6] Alaaeddine R., Elkhatib M.A., Mroueh A., Fouad H., Saad E.I., El-Sabban M.E., Plane F., El-Yazbi A.F. (2019). Impaired endothelium-dependent hyperpolarization underlies endothelial dysfunction during early metabolic challenge: Increased ROS generation and possible interference with NO function. J. Pharmacol. Exp. Ther..

[7] Mathew E., Mukkadan J. (2019). Clinical disparity of oxidative stress with blood pressure. J. Biomed. Sci..

[8] Kruger R., Schutte R., Huisman H., Van Rooyen J., Malan N., Fourie C., Louw R., Van der Westhuizen F., Van Deventer C., Malan L. (2012). Associations between reactive oxygen species, blood pressure and arterial stiffness in black South Africans: The SABPA study. J. Hum. Hypertens..

[9] Daliri E.B.-M., Lee B.H., Oh D.H. (2017). Current perspectives on antihypertensive probiotics. Probiotics Antimicrob..

[10] Daliri E.B.-M., Lee B.H., Oh D.H. (2018). Current trends and perspectives of bioactive peptides. Crit. Rev. Food Sci. Nutr..

[11] Dong J., Xu X., Liang Y., Head R., Bennett L. (2011). Inhibition of angiotensin converting enzyme activity by polyphenols from tea (*Camellia sinensis*) and links to processing method. Food Funct..

[12] Persson I.A.-L., Persson K., Andersson R.G. (2009). Effect of *Vaccinium myrtillus* and its polyphenols on angiotensin-converting enzyme activity in human endothelial cells. J. Agric. Food Chem..

[13] Al Shukor N., Van Camp J., Gonzales G.B., Staljanssens D., Struijs K., Zotti M.J., Raes K., Smagghe G. (2013). Angiotensin-converting enzyme inhibitory effects by plant phenolic compounds: A study of structure activity relationships. J. Agric. Food Chem..

[14] Kontani N., Omae R., Kagebayashi T., Kaneko K., Yamada Y., Mizushige T., Kanamoto R., Ohinata K. (2014). Characterization of Ile-His-Arg-Phe, a novel rice-derived vasorelaxing peptide with hypotensive and anorexigenic activities. Mol. Nutr. Food Res..

[15] Chen J., Liu S., Ye R., Cai G., Ji B., Wu Y. (2013). Angiotensin-I converting enzyme inhibitory tripeptides from rice protein hydrolysate: Purification and characterization. J. Funct. Foods.

[16] Pinciroli M., Aphalo P., Nardo A.E., Añón M.C., Quiroga A.V. (2019). Broken rice as a potential functional ingredient with inhibitory activity of renin and angiotensin-converting enzyme. Plant Foods Hum. Nutr..

[17] Aluko R.E. (2015). Antihypertensive peptides from food proteins. Annu. Rev. Food Sci. Technol..

[18] Oboh G., Ademiluyi A., Akindahunsi A. (2009). Changes in polyphenols distribution and antioxidant activity during fermentation of some underutilized legumes. Food Sci. Technol. Int..

[19] Rubak Y.T., Nuraida L., Iswantini D., Prangdimurti E. (2019). Production of antihypertensive bioactive peptides in fermented food by lactic acid bacteria—A review. Carpath. J. Food Sci. Technol..

[20] Zhao S., Zhao J., Bu D., Sun P., Wang J., Dong Z. (2014). Metabolomics analysis reveals large effect of roughage types on rumen microbial metabolic profile in dairy cows. Lett. Appl. Microbiol..

[21] Goldansaz S.A., Guo A.C., Sajed T., Steele M.A., Plastow G.S., Wishart D.S. (2017). Livestock metabolomics and the livestock metabolome: A systematic review. PLoS ONE.

[22] Yang Y., Dong G., Wang Z., Wang J., Zhang Z., Liu J. (2018). Rumen and plasma metabolomics profiling by UHPLC-QTOF/MS revealed metabolic alterations associated with a high-corn diet in beef steers. PLoS ONE.

[23] Metsalu T., Vilo J. (2015). ClustVis: A web tool for visualizing clustering of multivariate data using principal component analysis and heatmap. Nucleic Acids Res..

[24] Pinkard B.R., Gorman D.J., Rasmussen E.G., Maheshwari V., Kramlich J.C., Reinhall P.G., Novosselov I.V. (2020). Raman spectroscopic data from formic acid decomposition in subcritical and supercritical water. Data Brief.

[25] Tung L. (1966). Method of calculating molecular weight distribution function from gel permeation chromatograms. J. Appl. Polym. Sci..

[26] Berente B., De la Calle García D., Reichenbächer M., Danzer K. (2000). Method development for the determination of anthocyanins in red wines by high-performance liquid chromatography and classification of German red wines by means of multivariate statistical methods. J. Chromatogr. A.

[27] Zhou Z., Robards K., Helliwell S., Blanchard C. (2002). Composition and functional properties of rice. Int. J. Food Sci. Technol..

[28] Suwannapan O., Wachirattanapongmetee K., Thawornchinsombut S., Katekaew S. (2020). Angiotensin-I-converting enzyme (ACE)-inhibitory peptides from Thai jasmine rice bran protein hydrolysates. Int. J. Food Sci..

[29] Cirlini M., Ricci A., Galaverna G., Lazzi C. (2020). Application of lactic acid fermentation to elderberry juice: Changes in acidic and glucidic fractions. LWT Food Sci. Technol..

[30] Wang L., Zhu Q., Lu A., Liu X., Zhang L., Xu C., Liu X., Li H., Yang T. (2017). Sodium butyrate suppresses angiotensin II-induced hypertension by inhibition of renal (pro)renin receptor and intrarenal renin–angiotensin system. J. Hypertens..

[31] Bays H.E., Maccubbin D., Meehan A.G., Kuznetsova O., Mitchel Y.B., Paolini J.F. (2009). Blood pressure-lowering effects of extended-release niacin alone and extended-release niacin/laropiprant combination: A post hoc analysis of a 24-week, placebo-controlled trial in dyslipidemic patients. Clin. Ther..

[32] Dai T., Chen J., McClements D.J., Hu P., Ye X., Liu C., Li T. (2019). Protein–polyphenol interactions enhance the antioxidant capacity of phenolics: Analysis of rice glutelin–procyanidin dimer interactions. Food Func..

[33] Kumar S., Prahalathan P., Raja B. (2011). Antihypertensive and antioxidant potential of vanillic acid, a phenolic compound in L-NAME-induced hypertensive rats: A dose-dependence study. Redox Rep..

[34] Peixoto-Neves D., Wang Q., Leal-Cardoso J.H., Rossoni L.V., Jaggar J.H. (2015). Eugenol dilates mesenteric arteries and reduces systemic BP by activating endothelial cell TRPV 4 channels. Br. J. Pharmacol..

[35] Saravanakumar M., Raja B. (2011). Veratric acid, a phenolic acid attenuates blood pressure and oxidative stress in L-NAME induced hypertensive rats. Eur. J. Pharmacol..

[36] Jin L., Piao Z.H., Sun S., Liu B., Kim G.R., Seok Y.M., Lin M.Q., Ryu Y., Choi S.Y., Kee H.J. (2017). Gallic acid reduces blood pressure and attenuates oxidative stress and cardiac hypertrophy in spontaneously hypertensive rats. Sci. Rep..

[37] Luo D., Xu J., Chen X., Zhu X., Liu S., Li J., Xu X., Ma X., Zhao J., Ji X. (2020). (−)-Epigallocatechin-3-gallate (EGCG) attenuates salt-induced hypertension and renal injury in Dahl salt-sensitive rats. Sci. Rep..

[38] Sui H., Yan W. (2009). Effect of apigenin on SBP of spontaneous hypertension rats and its mechanism. J. Environ. Health.

[39] Yusuf M.A., Singh B.N., Sudheer S., Kharwar R.N., Siddiqui S., Abdel-Azeem A.M., Fernandes Fraceto L., Dashora K., Gupta V.K. (2019). Chrysophanol: A natural anthraquinone with multifaceted biotherapeutic potential. Biomolecules.

[40] Chandrasekara A., Shahidi F. (2012). Bioaccessibility and antioxidant potential of millet grain phenolics as affected by simulated in vitro digestion and microbial fermentation. J. Funct. Foods.

[41] Dey T.B., Kuhad R.C. (2014). Enhanced production and extraction of phenolic compounds from wheat by solid-state fermentation with *Rhizopus oryzae* RCK2012. Biotech. Rep..

[42] Liptáková D., Matejčeková Z., Valík L. (2017). Lactic acid bacteria and fermentation of cereals and pseudocereals. Ferment. Process..

[43] Wang Z.J., Liang C.L., Li G.M., Yu C.Y., Yin M. (2007). Stearic acid protects primary cultured cortical neurons against oxidative stress4. Acta Pharmacol. Sin..

[44] Alves N.F.B., de Queiroz T.M., de Almeida Travassos R., Magnani M., de Andrade Braga V. (2017). Acute treatment with lauric acid reduces blood pressure and oxidative stress in spontaneously hypertensive rats. Basic Clin. Pharmacol. Toxicol..

[45] Antony U., Sripriya G., Chandra T. (1996). The effect of fermentation on the primary nutrients in foxtail millet (*Setaria italica*). Food Chem..

[46] Umezawa H., Aoyagi T., Ogawa K., Naganawa H., Hamada M., Takeuchi T. (1984). Diprotins A and B, inhibitors of dipeptidyl aminopeptidase IV, produced by bacteria. J. Antibiot..

[47] Lan V.T.T., Ito K., Ohno M., Motoyama T., Ito S., Kawarasaki Y. (2015). Analyzing a dipeptide library to identify human dipeptidyl peptidase IV inhibitor. Food Chem..

[48] Cosenso-Martin L.N., Giollo-Junior L.T., Vilela-Martin J.F. (2015). DPP-4 inhibitor reduces central blood pressure in a diabetic and hypertensive patient: A case report. Medicine.

[49] Pacheco B.P., Crajoinas R.O., Couto G.K., Davel A.P.C., Lessa L.M., Rossoni L.V., Girardi A.C. (2011). Dipeptidyl peptidase IV inhibition attenuates blood pressure rising in young spontaneously hypertensive rats. Int. J. Hypertens..

[50] Zhang Y., Pechan T., Chang S.K.C. (2018). Antioxidant and angiotensin-I converting enzyme inhibitory activities of phenolic extracts and fractions derived from three phenolic-rich legume varieties. J. Funct. Foods.

[51] Santos M.C., Toson N.S.B., Pimentel M.C.B., Bordignon S.A.L., Mendez A.S.L., Henriques A.T. (2020). Polyphenols composition from leaves of *Cuphea* spp. and inhibitor potential, in vitro, of angiotensin I-converting enzyme (ACE). J. Ethnopharmacol..

[52] Wang X., Chen H., Fu X., Li S., Wei J. (2017). A novel antioxidant and ACE inhibitory peptide from rice bran protein: Biochemical characterization and molecular docking study. LWT Food Sci. Technol..

[53] Wang X., Wang J., Lin Y., Ding Y., Wang Y., Cheng X., Lin Z. (2011). QSAR study on angiotensin-converting enzyme inhibitor oligopeptides based on a novel set of sequence information descriptors. J. Mol. Model..

[54] Yano S., Suzuki K., Funatsu G. (1996). Isolation from α-zein of thermolysin peptides with angiotensin I-converting enzyme inhibitory activity. Biosci. Biotechnol. Biochem..

[55] Cheung H.-S., Wang F.-L., Ondetti M.A., Sabo E.F., Cushman D.W. (1980). Binding of peptide substrates and inhibitors of angiotensin-converting enzyme. Importance of the COOH-terminal dipeptide sequence. J. Biol. Chem..

[56] Zhou P., Yang C., Ren Y., Wang C., Tian F. (2013). What are the ideal properties for functional food peptides with antihypertensive effect? A computational peptidology approach. Food Chem..

[57] Hernández-Ledesma B., Recio I., Ramos M., Amigo L. (2002). Preparation of ovine and caprine β-lactoglobulin hydrolysates with ACE-inhibitory activity. Identification of active peptides from caprine β-lactoglobulin hydrolysed with thermolysin. Int. Dairy J..

[58] Castellano P., Aristoy M.-C., Sentandreu M.Á., Vignolo G., Toldrá F. (2013). Peptides with angiotensin I converting enzyme (ACE) inhibitory activity generated from porcine skeletal muscle proteins by the action of meat-borne Lactobacillus. J. Proteom..

[59] Wu H., He H.-L., Chen X.-L., Sun C.-Y., Zhang Y.-Z., Zhou B.-C. (2008). Purification and identification of novel angiotensin-I-converting enzyme inhibitory peptides from shark meat hydrolysate. Process Biochem..

[60] Ichimura T., Hu J., Aita D.Q., Maruyama S. (2003). Angiotensin I-converting enzyme inhibitory activity and insulin secretion stimulative activity of fermented fish sauce. J. Biosci. Bioeng..

[61] Byun H.-G., Kim S.-K. (2002). Structure and activity of angiotensin I converting enzyme inhibitory peptides derived from Alaskan pollack skin. BMB Rep..

[62] Suetsuna K., Ukeda H., Ochi H. (2000). Isolation and characterization of free radical scavenging activities peptides derived from casein. J. Nutr. Biochem..

[63] Kohmura M., Nio N., Kubo K., Minoshima Y., Munekata E., Ariyoshi Y. (1989). Inhibition of angiotensin-converting enzyme by synthetic peptides of human β-casein. Agric. Biol. Chem..

[64] Gu Y., Majumder K., Wu J. (2011). QSAR-aided in silico approach in evaluation of food proteins as precursors of ACE inhibitory peptides. Food Res. Int..

[65] Tauzin J., Miclo L., Gaillard J.-L. (2002). Angiotensin-I-converting enzyme inhibitory peptides from tryptic hydrolysate of bovine αS2-casein. FEBS Lett..

[66] Nogata Y., Nagamine T., Yanaka M., Ohta H. (2009). Angiotensin I converting enzyme inhibitory peptides produced by autolysis reactions from wheat bran. J. Agric. Food Chem..

[67] Escudero E., Sentandreu M.A., Arihara K., Toldra F. (2010). Angiotensin I-converting enzyme inhibitory peptides generated from in vitro gastrointestinal digestion of pork meat. J. Agric. Food Chem..

[68] He R., Malomo S.A., Girgih A.T., Ju X., Aluko R.E. (2013). Glycinyl-histidinyl-serine (GHS), a novel rapeseed protein-derived peptide has blood pressure-lowering effect in spontaneously hypertensive rats. J. Agric. Food Chem..

[69] Nakahara T., Sano A., Yamaguchi H., Sugimoto K., Chikata H., Kinoshita E., Uchida R. (2010). Antihypertensive effect of peptide-enriched soy sauce-like seasoning and identification of its angiotensin I-converting enzyme inhibitory substances. J. Agric. Food Chem..

[70] Del Mar Contreras M., Carrón R., Montero M.J., Ramos M., Recio I. (2009). Novel casein-derived peptides with antihypertensive activity. Int. Dairy J..

[71] Minervini F., Algaron F., Rizzello C., Fox P., Monnet V., Gobbetti M. (2003). Angiotensin I-converting-enzyme-inhibitory and antibacterial peptides from *Lactobacillus helveticus* PR4 proteinase-hydrolyzed caseins of milk from six species. Appl. Environ. Microbiol..

[72] Udenigwe C.C., Adebiyi A.P., Doyen A., Li H., Bazinet L., Aluko R.E. (2012). Low molecular weight flaxseed protein-derived arginine-containing peptides reduced blood pressure of spontaneously hypertensive rats faster than amino acid form of arginine and native flaxseed protein. Food Chem..

[73] Wu J., Aluko R.E., Nakai S. (2006). Structural requirements of angiotensin I-converting enzyme inhibitory peptides: Quantitative structure− activity relationship study of di-and tripeptides. J. Agric. Food Chem..

