# Supplementary material for: Untargeted Metabolomics of Fermented Rice Using UHPLC Q-TOF MS/MS Reveals an Abundance of Potential Antihypertensive Compounds
Source: Foods. 2020 Jul 27;9(8):1007. doi: 10.3390/foods9081007 (PMC7466378; doi:10.3390/foods9081007)
Supplement: Supplementary file 1 [file foods-09-01007-s001.pdf]

## Supplementary data

**Table S1.** Procedure for assay of ACE activity.

| | Test samples | Positive control | Negative control |
|---|---|---|---|
| ACE-1 positive control (μl) | 10 | 10 | 10 |
| ACE1 assay buffer (μl) | 40 | 40 | 60 |
| Inhibitor (μL) | 20 | 20 | 0 |
| ACE-1 substrate (μl) | 50 | 50 | 50 |

**Table S2.** Organic acids and volatile compounds in destarched rice, Prozyme treated destarched rice (DP) and fermented DP.

| Retention Time (min) | Adduct / Charge | Peak area | | | Precursor mass | Found at mass | Formula finder results | Organic acids |
|---|---|---|---|---|---|---|---|---|
| | | Destarched rice | Destarched rice + Prozyme (DP) | Fermented DP | | | | |
| 1 | [M-H]- | 20840000 | 1.80E+07 | 2.01E+07 | 195.052 | 195.0511 | C6H12O7 | Gluconic acid |
| 1.01 | [M-H]- | 328000 | 3.31E+05 | 0 | 209.031 | 209.0307 | C6H10O8 | Glucaric acid |
| 1.17 | [M-H]- | 151700 | 2.39E+05 | 1.96E+06 | 133.015 | 133.0143 | C4H6O5 | Malic acid |
| 1.49 | [M-H]- | 64250 | 8.04E+04 | 5.62E+05 | 111.01 | 111.0089 | C5H4O3 | Furoic acid |
| 1.79 | [M-H]- | 178600 | 1.83E+05 | 0.00E+00 | 117.02 | 117.0194 | C4H6O4 | Succinic acid |
| 2.44 | [M-H]- | 4298 | 6.29E+03 | 1.17E+06 | 103.041 | 103.0401 | C4H8O3 | Hydroxybutanoic acid |
| 3.35 | [M-H]- | 638700 | 6.42E+05 | 6.43E+05 | 205.036 | 205.0356 | C7H10O7 | Homocitric acid |
| 5.97 | [M-H]- | 803100 | 2.31E+05 | 5.76E+05 | 218.104 | 218.1036 | C9H17NO5 | Pantothenic acid |
| 8.65 | [M-H]- | 211100 | 2.16E+05 | 1.20E+05 | 259.077 | 259.0761 | C18H12O2 | Pyreneacetic acid |
| 9.95 | [M-H]- | 329500 | 2.88E+05 | 3.18E+05 | 191.021 | 191.0191 | C6H8O7 | Citric acid |
| 10.01 | [M-H]- | 0 | 0.00E+00 | 4.78E+04 | 355.068 | 355.0656 | C12H8N10O4 | Binicotinic acid |
| 11.3 | [M-H]- | 0 | 2.74E+05 | 2.21E+05 | 157.051 | 157.0512 | C7H10O4 | Dioxoheptanoic acid |
| 11.56 | [M-H]- | 0 | 0.00E+00 | 3.91E+02 | 131.072 | 131.0713 | C6H12O3 | Butoxyacetic acid |
| 12.96 | [M-H]- | 0 | 0.00E+00 | 7.74E+02 | 173.083 | 173.0821 | C8H14O4 | Suberic acid |
| 13.33 | [M-H]- | 0 | 0.00E+00 | 3.99E+03 | 237.041 | 237.0406 | C11H10O6 | Oxobutyric acid |
| 15.81 | [M-H]- | 0 | 3.98E+05 | 3.49E+05 | 201.114 | 201.1134 | C10H18O4 | Sebacic acid |
| 17.27 | [M-H]- | 0 | 0.00E+00 | 1.31E+04 | 327.219 | 327.2178 | C18H32O5 | Malyngic acid |
| 18.55 | [M-H]- | 0 | 0.00E+00 | 6.41E+05 | 333.131 | 333.13 | C13H22N2O8 | Pseudaminic acid |
| 19.2 | [M-H]- | 0 | 1.84E+05 | 5.15E+04 | 154.975 | 154.9742 | C5H4N2S2 | Pyrazinoic acid |
| 19.62 | [M-H]- | 0 | 2.92E+05 | 3.47E+05 | 297.208 | 297.2073 | C17H30O4 | Butanedioic acid |

**Table S3.** Amino acids in destarched rice, Prozyme treated destarched rice (DP) and fermented DP.

| Retention time (min) | Adduct / Charge | Peak area | | | Precursor mass | Found at mass | Formula finder results | Amino acids |
|---|---|---|---|---|---|---|---|---|
| | | Destarched rice | Destarched rice + Prozyme (DP) | Fermented DP | | | | |
| 0.89 | [M-H]- | 6573 | 4.05E+05 | 514400 | 131.084 | 131.0827 | C5H12N2O2 | Ornithine |
| 0.89 | [M-H]- | 31680 | 1.57E+06 | 1159000 | 154.063 | 154.0624 | C6H9N3O2 | Histidine |
| 0.95 | [M-H]- | 36870 | 0 | 1681000 | 104.036 | 104.0354 | C3H7NO3 | Serine |
| 0.97 | [M-H]- | 183500 | 5.81E+05 | 501000 | 131.047 | 131.0463 | C4H8N2O3 | Asparagine |
| 0.97 | [M-H]- | 597800 | 1.06E+06 | 623200 | 132.031 | 132.0303 | C4H7NO4 | Aspartic acid |
| 0.97 | [M-H]- | 278200 | 1.57E+06 | 2685000 | 102.057 | 102.0561 | C4H9NO2 | $\alpha$-Aminobutyric acid |
| 0.97 | [M-H]- | 105800 | 4.15E+05 | 703500 | 146.047 | 146.0459 | C5H9NO4 | Glutamic acid |
| 0.97 | [M-H]- | 530100 | 2.45E+06 | 4215000 | 145.063 | 145.0619 | C5H10N2O3 | Glutamine |
| 0.98 | [M-H]- | 154100 | 7.95E+05 | 415600 | 118.052 | 118.051 | C4H9NO3 | Threonine |
| 1.26 | [M-H]- | 44610 | 3.12E+06 | 4225000 | 148.045 | 148.0441 | C5H11NO2S | Methionine |
| 1.55 | [M-H]- | 95130 | 6.26E+06 | 2249000 | 128.036 | 128.0354 | C5H7NO3 | Pyroglutamic acid |
| 1.76 | [M-H]- | 115300 | 0 | 0 | 130.088 | 130.0874 | C6H13NO2 | Leucine |
| 3.13 | [M-H]- | 3404 | 7.80E+03 | 0 | 164.073 | 164.0717 | C9H11NO2 | Phenylalanine |
| 6.03 | [M-H]- | 47720 | 2.57E+06 | 0 | 203.084 | 203.0828 | C11H12N2O2 | Tryptophan |
| 12.27 | [M-H]- | 283200 | 0 | 0 | 339.106 | 339.1052 | C12H16N6O6 | Arginine |
| 14.89 | [M-H]- | 0 | 8.16E+05 | 5.43E+05 | 269.047 | 269.0444 | C7H14N2O7S | Cysteic acid |

**Table S4.** Phenolic compounds in destarched rice, Prozyme treated destarched rice (DP) and fermented DP.

| Retention Time (min) | Adduct / Charge | Peak area | | | Precursor Mass | Found At Mass | Formula Finder Results | Phenolic compounds |
|---|---|---|---|---|---|---|---|---|
| | | Destarched rice | Destarched rice + Prozyme (DP) | Fermented DP | | | | |
| 3.16 | [M-H]- | 519400 | 0 | 0 | 147.046 | 147.0453 | C9H8O2 | Cinnamic acid |
| 8.23 | [M-H]- | 164500 | 1.95E+05 | 1.51E+04 | 181.052 | 181.0507 | C14H22O2 | Veratric acid |
| 10.37 | [M-H]- | 130500 | 1.23E+05 | 1.28E+05 | 167.036 | 167.0349 | C8H8O4 | Vanillic acid |
| 11.15 | [M-H]- | 13660 | 1.23E+06 | 1.17E+06 | 197.047 | 197.0453 | C9H10O5 | Ethyl gallate |
| 11.17 | [M-H]- | 18370 | 2.04E+04 | 1.59E+07 | 197.047 | 197.0453 | C9H10O5 | Syringic acid |
| 11.17 | [M-H]- | 8533 | 0 | 0.00E+00 | 163.077 | 163.0765 | C10H12O2 | Eugenol |
| 12 | [M-H]- | 431300 | 3.76E+05 | 4.77E+05 | 163.041 | 163.04 | C9H8O3 | Coumarinic acid |
| 12.34 | [M-H]- | 718600 | 8.02E+05 | 7.85E+05 | 305.071 | 305.067 | C15H14O7 | Epigallocatechin |
| 12.59 | [M-H]- | 68830 | 3.80E+05 | 1.68E+05 | 193.052 | 193.0508 | C10H10O4 | Ferulic acid |
| 14.3 | [M-H]- | 0 | 0 | 6.40E+05 | 269.047 | 269.0456 | C15H10O5 | Apigenin |
| 14.95 | [M-H]- | 0 | 2.60E+02 | 1.98E+06 | 253.052 | 253.0509 | C15H10O4 | Chrysophanol |
| 15.35 | [M-H]- | 0 | 0 | 7.48E+03 | 301.036 | 301.0357 | C15H10O7 | Quercetin |
| 17.8 | [M-H]- | 0 | 0 | 7.85E+03 | 431.173 | 431.1715 | C23H28O8 | Flavaspidic acid PB |
| 18.19 | [M-H]- | 0 | 0 | 6.27E+04 | 309.172 | 309.1709 | C17H26O5 | Dodecyl gallate |
| 19.71 | [M-H]- | 0 | 0 | 2.89E+04 | 277.182 | 277.1809 | C10H12O2 | Paradol |
| 20.17 | [M-H]- | 0 | 6.60E+05 | 6.64E+05 | 221.156 | 221.1547 | C14H22O2 | 3,5-Di-tert-butylphenol |
| 20.17 | [M-H]- | 56110 | 0 | 1.86E+06 | 173.01 | 173.0093 | C14H22O2 | Benzenehexol |
